# ﻿Three new species of the genus *Weintrauboa* Hormiga, 2003 (Araneae, Linyphiidae) from China

**DOI:** 10.3897/zookeys.1236.146324

**Published:** 2025-04-29

**Authors:** Zhizhong Gao, Muhammad Irfan, Lu-Yu Wang

**Affiliations:** 1 Department of Biology, Xinzhou Normal University, Xinzhou, Shanxi 034000, China; 2 Key Laboratory of Eco-environments in Three Gorges Reservoir Region (Ministry of Education), School of Life Sciences, Southwest University, Chongqing 400715, China; 3 College of Plant Protection, Southwest University, Chongqing 400715, China

**Keywords:** Description, distribution, morphology, sheet-web spiders, taxonomy

## Abstract

Three new species of the genus *Weintrauboa* Hormiga, 2003 are described here as: *W.denticulata***sp. nov.** (Hunan, ♂), *W.shenwu***sp. nov.** (Hubei and Chongqing, ♂♀), and *W.wanglangensis***sp. nov.** (Sichuan, ♂♀). Detailed descriptions, photographs of genital characters, somatic features, and a distribution map are provided.

## ﻿Introduction

Linyphiidae is one of the most diverse spider families worldwide, comprising 640 extant genera and 4947 species globally, including 11 fossil genera and 62 species (WSC 2025). In China, approximately 608 species across 182 genera have been documented (Tanasevitch 2025). The genus *Weintrauboa* Hormiga, 2003 includes eight species found in China (Guizhou, Sichuan, Yunnan), Japan, and Russia (Far East, Sakhalin) (WSC 2025). Initially classified within the family Pimoidae Wunderlich, 1986, the genus was transferred to Linyphiidae based on a molecular analysis and reinterpretation of its morphology (Hormiga et al. 2021).

Recent studies of linyphiid spiders have mainly focused on the southern regions of the country: Yunnan Province (Zhao and Li 2014; Irfan and Peng 2018, 2019a, 2019b; Zhou et al. 2018, 2021, 2023; Irfan et al. 2019, 2020, 2021, 2022a, 2022b, 2023a, 2023b, 2024, 2025; Zhang et al. 2022; Yang et al. 2023) and Chongqing Region (Irfan et al. 2023a, 2023b). These studies have not only substantially increased the known diversity of Linyphiidae in Yunnan and Chongqing but also suggest that a significant number of species remain undiscovered in southern China. Future extensive research in this region is likely to reveal more species, further enriching our understanding of this complex and diverse spider family. While examining specimens collected from south China, three new species of the genus *Weintrauboa* were identified and are described here.

## ﻿Material and methods

Specimens were collected by hand picking and sieving leaf litter, and were kept in 75% ethanol. The left male palps were used for photography. After dissection, epigynes were cleared in trypsin enzyme solution before examination and photography. All specimens were examined, photographed and measured using a Leica M205A stereomicroscope and LAS00 software (ver. 4.6). Left male palps and epigynes were examined and photographed after dissection. Compound focus images were generated using Helicon Focus ver. 6.7.1 software. Eye sizes were measured at the maximum dorsal diameter. Legs measurements are shown as total length (femur, patella, tibia, metatarsus, tarsus). All measurements are in millimeters. Specimens are deposited in the School of Life Sciences, Southwest University, Chongqing (SWUC), China. The map was created using the online mapping software SimpleMappr (Shorthouse 2010) (Fig. 7). The terminology used in the text and figure legends follows Hormiga et al. (2021). In the text, “Fig.” and “Figs” refer to figures herein, while “fig.” and “figs” refer to figures published elsewhere.

The following abbreviations are used in the text and figures: **a.s.l.** = above sea level; **AER** = anterior eye row; **ALE** = anterior lateral eyes; **AME** = anterior median eyes; **AME–ALE** = the distance between AME and ALE; **AME–AME** = the distance between AMEs; **ARP** = anterior radical process; **CD** = copulatory ducts; **CO** = copulatory openings; **CP** = cymbial process (CDP in Hormiga 1994); **DP** = dorsal plate; **E** = embolus; **EF** = embolus flap; **FD** = fertilization ducts; **PC** = paracymbium; **PER** = posterior eye row; **PLE** = posterior lateral eyes; **PME** = posterior median eyes; **PME–PLE** = distance between PME and PLE; **PME–PME** = distance between PMEs; **S** = spermatheca; **SPT** = suprategulum; **ST** = subtegulum; **T** = tegulum; **TmI** = position of trichobothrium on metatarsus I; **VP** = ventral plate.

## ﻿Taxonomy

### ﻿Family Linyphiidae Blackwall, 1859

#### ﻿Genus Weintrauboa

Taxon classificationAnimaliaAraneaeLinyphiidae

Hormiga, 2003

B5BB41AD-D936-50B5-B3B6-73E54A313B5F

##### Type species.

*Labullacontortipes* Karsch, 1881; gender feminine.

#### ﻿ Weintrauboa denticulata sp. nov.

Taxon classificationAnimaliaAraneaeLinyphiidae

1392343E-41A7-56BC-9C4E-12492CE321F9

https://zoobank.org/131F061C-4553-4D40-9306-4741C5DEF9D2

**Figure 1.**
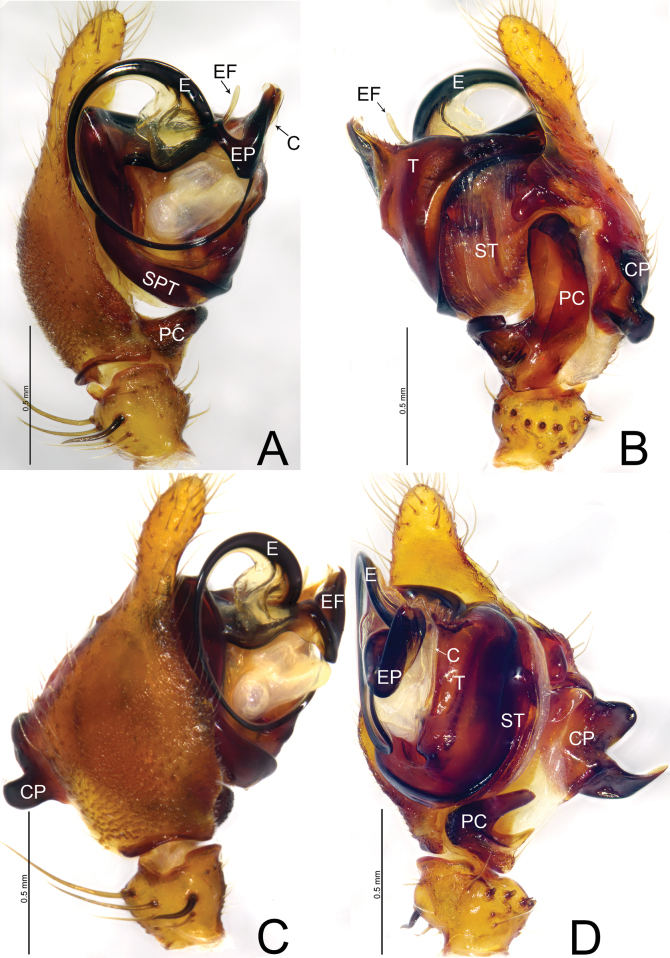
*Weintrauboadenticulata* sp. nov., male holotype **A** palp, prolateral view **B** palp, retrolateral view **C** palp, dorsal view **D** palp, ventral view. Abbreviations: CP = cymbial process; E = embolus; EF = embolus flap; EP = embolus process; PC = paracymbium; SPT = suprategulum; ST = subtegulum; T = tegulum.

Figs 1 , 6A , 7 (齿文蛛)

##### Type material.

***Holotype*** ♂ (SWUC-T-LIN-38-01); China, Hunan Province, Changsha City, Yuelu District, Yuelu Mountain, 28°11'33.9"N, 112°56'17.52"E, a.s.l. 208 m, 27.IX.2017, Wang Luyu leg.

##### Etymology.

The specific epithet is derived from Latin adjective “*denticulatus*” meaning “toothed”, referring to the tegulum apically toothed in the male palp.

##### Diagnosis.

This new species resembles *Weintrauboayele* Hormiga, 2008 in having similar embolus in male palp (Fig. 1A–D; Hormiga 2008, figs 2A–C, 3A–C; Hormiga et al. 2021, fig. 6A, B), but can be differentiated by the tegular apophysis with teeth in *W.denticulata* sp. nov. (Fig. 1B, D; vs teeth absent); embolic process tip grooved in ventral view in *W.denticulata* sp. nov. (Fig. 1D; vs hook-shaped); embolic flap like in retrolateral view in *W.denticulata* sp. nov. (Fig. 1B; vs almost wing-shaped); proximal ramus of cymbial process two times longer than distal one in *W.denticulata* sp. nov. (Fig. 1D; vs both rami almost same in length).

##### Description.

**Male** (holotype, Fig. 6A) total length 5.63. Carapace 2.86 long, 2.23 wide; opisthosoma 2.77 long, 2.13 wide. Eye sizes and interdistances: AME 0.17, ALE 0.16, PME 0.17, PLE 0.14; AME–AME 0.10, AME–ALE 0.09, PME–PME 0.14, PME–PLE 0.16, ALE–PLE contiguous. MOA 0.91 long, front width 0.62, back width 0.51. Clypeus height 0.27. Chelicerae brown, with three promarginal and three retromarginal teeth. Leg measurements: I 11.6 (3.08, 1.11, 2.58, 3.23, 1.60); II 10.96 (2.95, 1.02, 2.53, 3.12, 1.34); III 8.41 (2.47, 0.79, 1.84, 2.32, 0.99); IV 10.26 (2.88, 0.88, 2.45, 2.88, 1.17). Leg formula: 1243.

***Palp*** (Fig. 1A–D). Patella as long as tibia, ventrally grooved, dorsally with long thick spine. Tibia cone-shaped, with one retrolateral trichobothrium, retrolateral margin with seven thick spines. Cymbium with an ectal process wider than long, with bifurcated tip, proximal ramus hook-shaped, two times longer than distal one with pointed end, distal ramus tongue-shaped with blunt end; retrolateral margin of cymbium with tongue-shaped projection extending ventrally with blunt tip. Paracymbium bowl-shaped, apically hook-shaped, with median margin edge curved inward. Tegulum large, apically with long tegular apophysis with seven teeth, tip membranous. Conductor membranous, as long as patella, present on apical end of tegulum. Embolus circular with fine tip, embolic flap rod-like slightly curved with blunt tip, embolic process sclerotized, apically tip grooved, parallel to tegular apophysis.

**Female.** Unknown.

##### Distribution.

Known only from the type locality, Hunan, China (Fig. 7).

#### ﻿ Weintrauboa shenwu sp. nov.

Taxon classificationAnimaliaAraneaeLinyphiidae

4558321B-11EC-5E14-9771-FFFC42C6EFD5

https://zoobank.org/9A89FD67-41A6-4424-8CE9-CD19A91DF5DE

**Figure 2.**
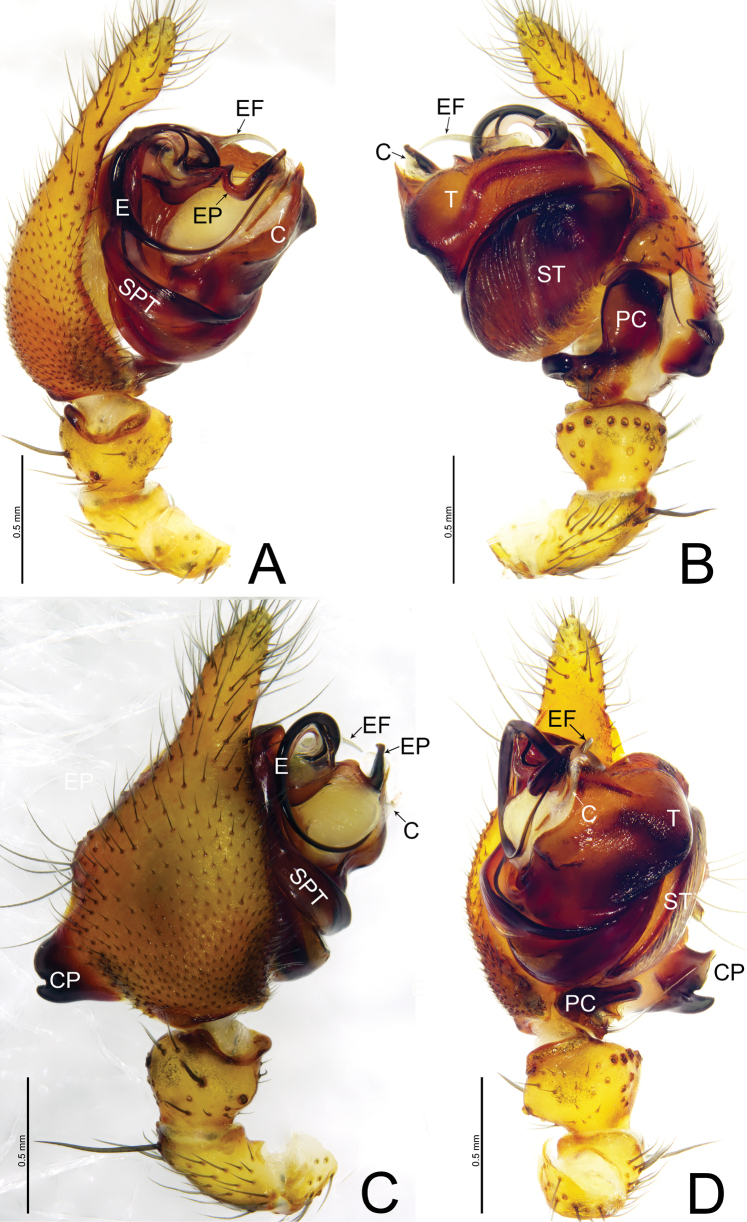
*Weintrauboashennongjiaensis* sp. nov., male holotype **A** palp, prolateral view **B** palp, retrolateral view **C** palp, dorsal view **D** palp, ventral view. Abbreviations: CP = cymbial process; E = embolus; EF = embolus flap; EP = embolus process; PC = paracymbium; SPT = suprategulum; ST = subtegulum; T = tegulum.

**Figure 3.**
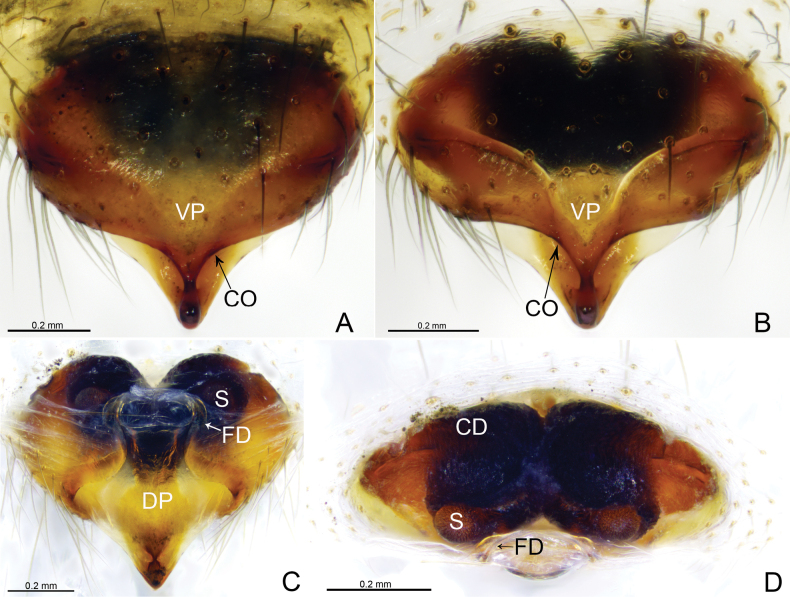
*Weintrauboashennongjiaensis* sp. nov., female paratype **A, B** epigyne, ventral view **C** vulva, dorsal view **D** vulva, anterior view. Abbreviations: CD = copulatory duct; CO = copulatory opening; DP = dorsal plate; FD = fertilization duct; S = spermathecae; VP = ventral plate.

Figs 2 , 3 , 6A, B , 7 (神巫文蛛)

##### Type material.

***Holotype*** ♂ (SWUC-T-LIN-39-01): China, Hubei Province, Shennongjia, Yazikou, 31°30'55.0008"N, 110°19'58.0008"E, 1817 m a.s.l., 24.X.2020, L.Y. Wang, Y. Zhang, J.X. Zhao and J.S. Luo leg. ***Paratypes***: 1♀ (SWUC-T-LIN-39-02), with same data as holotype • 2♂2♀ (SWUC-T-LIN-39-03~06), Hubei Province, Shennongjia, Hongping Town, 31°31'27.9957"N, 110°20'9.0416"E, 1711 m a.s.l., 14.VI.2023, Z.S. Zhang, X.L. Chen and Q.L. Lu leg. • **Chongqing Municipality**: 1♀ (SWUC-T-LIN-39-07), Wushan County, Dangyang Town, Xiejiacao, 31°26'57.00"N, 109°58'45.57"E, a.s.l. 1449 m, 02.X.2021, L.Y. Wang, T.Y. Ren, J.X. Zhao, L. Xiao and X.W. Zhou leg. • 5♂8♀ (SWUC-T-LIN-39-08~20), Wushan County, Guanyang Town, Pingqian, 31°22'22.75"N, 109°56'17.25"E, a.s.l. 1832 m, 04.X.2021, L.Y. Wang, T.Y. Ren, J.X. Zhao, L. Xiao and X.W. Zhou leg.

##### Etymology.

The specific name is derived from the Chinese word ‘shen’ and ‘wu’; Shen is the first name for Shennongjia and Wu is an abbreviated name for Wushan; noun in apposition.

##### Diagnosis.

**Figure 4.**
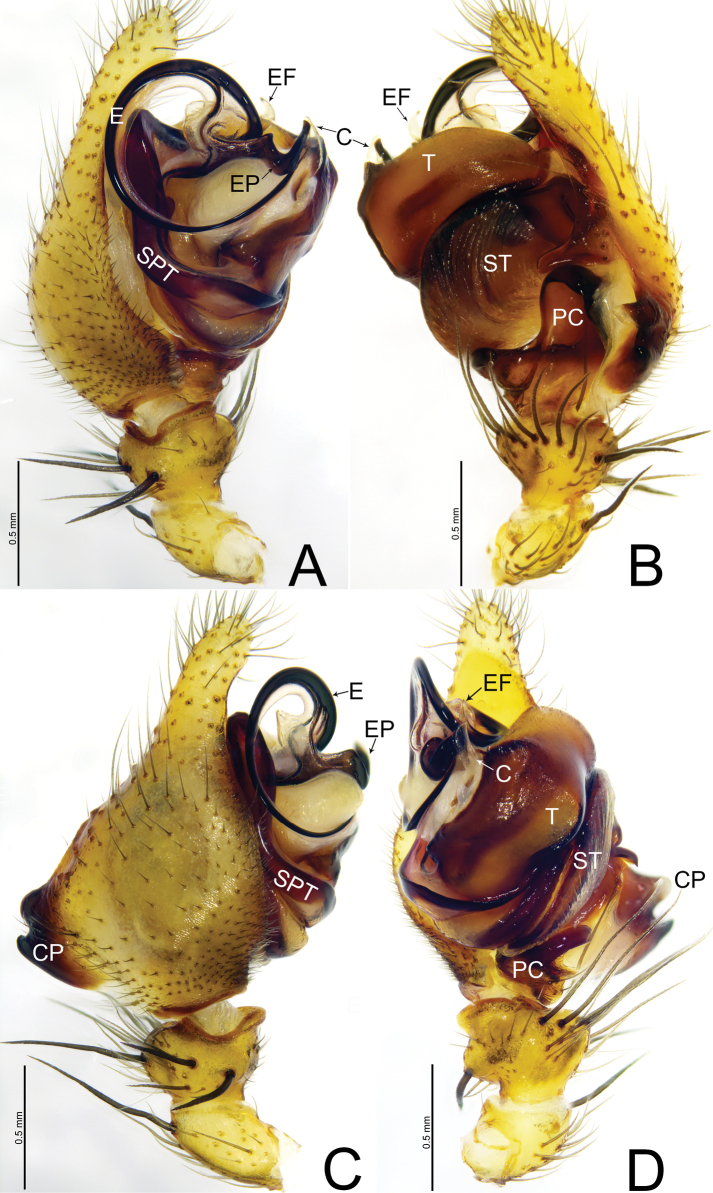
*Weintrauboawanglangensis* sp. nov., male holotype **A** palp, prolateral view **B** palp, retrolateral view **C** palp, dorsal view **D** palp, ventral view. Abbreviations: CP = cymbial process; E = embolus; EF = embolus flap; EP = embolus process; PC = paracymbium; SPT = suprategulum; ST = subtegulum; T = tegulum.

**Figure 5.**
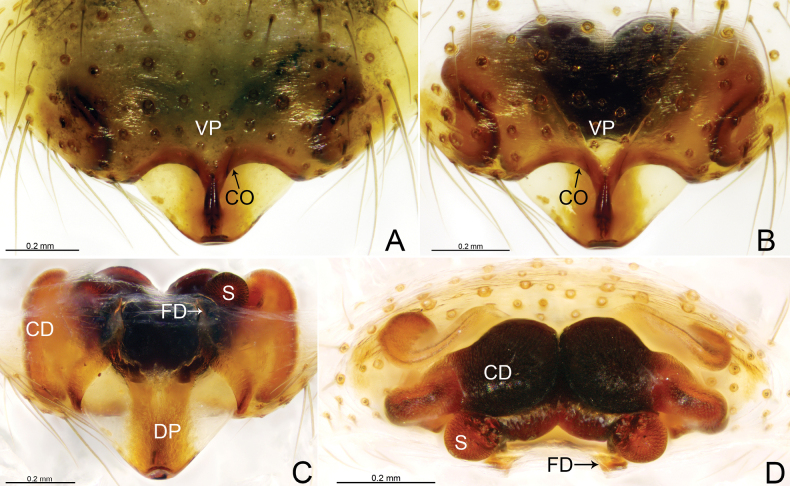
*Weintrauboawanglangensis* sp. nov., female paratype **A, B** epigyne, ventral view **C** vulva, dorsal view **D** vulva, anterior view. Abbreviations: CD = copulatory duct; CO = copulatory opening; DP = dorsal plate; FD = fertilization duct; S = spermathecae; VP = ventral plate.

*Weintrauboashenwu* resembles those of *W.wanglangensis* and *W.yele* Hormiga, 2008 in having a similar embolus and embolic process in male palp (Figs 2A–D, 4A–D; Hormiga 2008, figs 2A–C, 3A–C; Hormiga et al. 2021, fig. 6A–B) and can be distinguished by the embolic flap needle-shaped in *W.shenwu* (Fig. 2A, B; vs horn-shaped in *W.wanglangensis* and wing-shaped in *W.yele*); distal ramus of cymbial process somewhat rectangular in ventral view in *W.shenwu* (Fig. 2A, B; vs somewhat thumb-shaped both in *W.wanglangensis* and *W.yele*). Females of *W.shenwu* resemble *W.wanglangensis* in having similar morphology of epigyne (Figs 3A–D, 5A–D), but can be distinguished by the copulatory duct comma-shaped in *W.shenwu* (Fig. 3A–C; vs sinuous with three loops before entering spermathecae in *W.wanglangensis*, Fig. 5A–D); dorsal plate posteriorly triangular in *W.shenwu* (Fig. 3A–C; vs trapezoid in *W.wanglangensis*, Fig. 5A–D).

##### Description.

**Figure 6.**
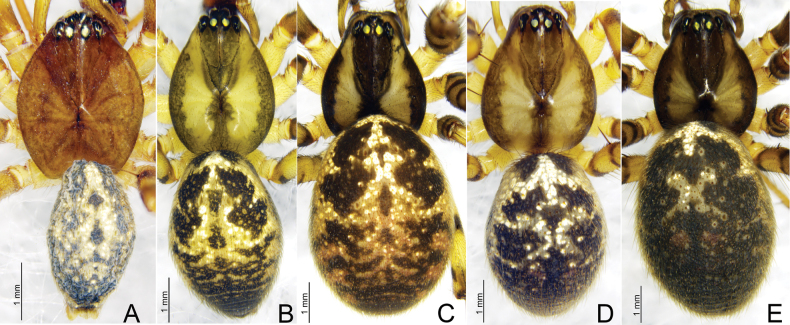
*Weintrauboa* species habitus, dorsal view **A***W.denticulata* sp. nov. male holotype **B, C***W.shennongjiaensis* sp. nov., male holotype (**B**), female paratype (**C**) **D, E***W.wanglangensis* sp. nov., male holotype (**D**) female paratype (**E**).

**Male** (holotype, Fig. 6B) total length 7.01. Carapace 3.42 long, 2.39 wide; opisthosoma 3.81 long, 2.55 wide. Eye sizes and interdistances: AME 0.18, ALE 0.22, PME 0.18, PLE 0.18; AME–AME 0.09, AME–ALE 0.11, PME–PME 0.12, PME–PLE 0.17, ALE–PLE 0.02. MOA 0.49 long, front width 0.47, back width 0.49. Clypeus height 0.46. Chelicerae brown, with four promarginal and three retromarginal teeth. Leg measurements: I 21.41 (5.55, 7.19, 6.08, 2.59); II 16.96 (4.63, 5.37, 4.79, 2.17); III 11.15 (3.27, 3.46, 2.93, 1.49); IV 13.44 (3.72, 4.07, 3.69, 1.96). Leg formula: 1243.

***Palp*** (Fig. 2A–D). Patella as long as tibia, ventrally grooved, dorsally with long thick spine. Tibia cone-shaped, with one retrolateral and one dorsal trichobothrium, retrolateral margin with eight thick spines. Cymbium with an ectal process wider than long, half the length of tibia, with bifurcated tip, proximal ramus thumb-shaped and distal ramus somewhat rectangular; retrolateral margin of cymbium with thumb-shaped projection extending ventrally with blunt tip. Paracymbium bowl-shaped, apically hook-shaped, with median margin edge curved inward. Tegulum large, pointed apically. Distal suprategular apophysis sclerotized reduced. Conductor small, membranous, present on apical end of tegulum. Embolus circular with fine tip, embolic flap needle-shaped, slightly curved with pointed tip, embolic process sclerotized, apically expanded with blunt tip, extending towards ventral side of tegulum.

**Female** (paratype, Fig. 6C) total length 7.62. Prosoma 3.32 long, 2.63 wide; opisthosoma 5.05 long, 3.64 wide. Eye sizes and interdistances: AME 0.21, ALE 0.23, PME 0.19, PLE 0.20; AME–AME 0.05, AME–ALE 0.09, PME–PME 0.10, PME–PLE 0.16. ALE–PLE 0.03. MOA 0.56 long, front width 0.44, back width 0.49. Clypeus height 0.38. Leg measurements: I 13.60 (3.67, 4.34, 3.52, 2.07); II 12.12 (3.41, 3.89, 3.03, 1.79); III 9.54 (2.83, 2.87, 2.43, 1.41); IV 11.68 (3.40, 3.69, 3.05, 1.54). Leg formula: 1243.

***Epigyne*** (Fig. 3A–D). Epigynal plate 1.5 times wider than long. Most of the atrium divided by septum. Ventral plate oval, anteriorly grooved, posterior margin convex. Copulatory openings present within atrium. Dorsal plate somewhat triangular extending posteriorly. Copulatory ducts elongated, V-shaped in ventral view, forming broad loop extending anteriorly before entering spermathecae. Spermathecae round, separated by distance equal to four times their diameter. Fertilization ducts present mesally.

##### Variation.

Males (*N* = 2) total length 5.85–7.01; females (*N* = 2) total length 7.06–8.03.

##### Distribution.

**Figure 7.**
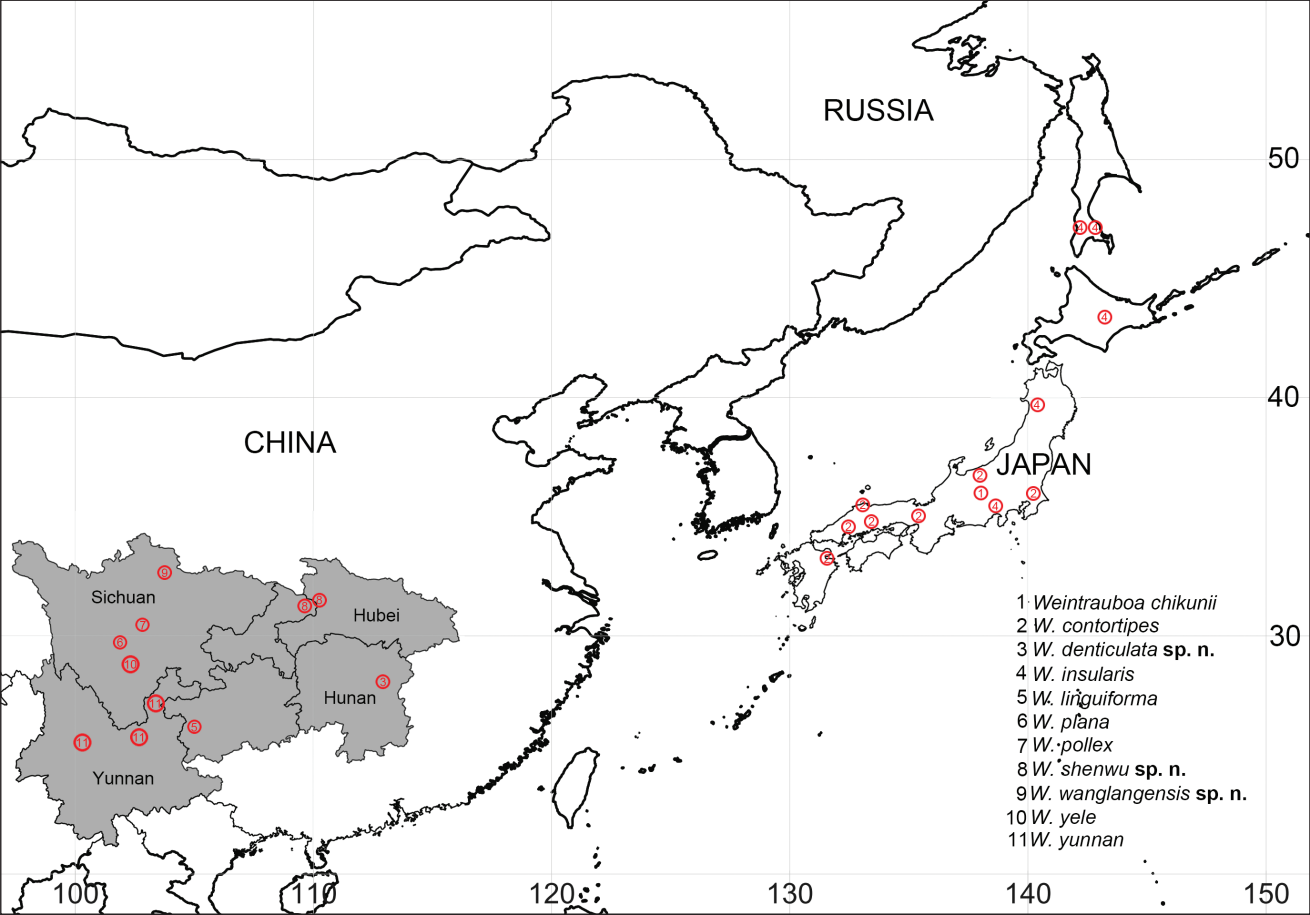
Distribution of *Weintrauboa* species (WSC 2025).

China (Hubei, Chongqing) (Fig. 7).

#### ﻿ Weintrauboa wanglangensis sp. nov.

Taxon classificationAnimaliaAraneaeLinyphiidae

AF97ABC0-BADE-50C9-8B66-909BAE848ACE

https://zoobank.org/8B70618A-53F1-4657-8B3D-EFBAF3966CE8

Figs 4 , 5 , 6D, E , 7 (王朗文蛛)

##### Type material.

***Holotype*** ♂ (SWUC-T-LIN-40-01): China, Sichuan Province, Pingwu County, Wanglang National Nature Reserve, Wuyangchang, 32°58'3.8388"N, 104°6'17.9388"E, a.s.l. 2503 m, 24.IX.2019, L.Y. Wang, P. Liu, T. Yuan, Z. Fan, Y. Zhang and M. Zhang leg. ***Paratypes***: 22♂15♀, same data as holotype (SWUC-T-LIN-40-02~38).

##### Etymology.

The specific epithet is derived from the type locality; adjective.

##### Diagnosis.

See diagnosis of *Weintrauboashenwu* sp. nov.

##### Description.

**Male** (holotype, Fig. 6D) total length 7.64. Carapace 3.53 long, 2.65 wide; opisthosoma 4.21 long, 2.68 wide. Eye sizes and interdistances: AME 0.22, ALE 0.22, PME 0.18, PLE 0.20; AME–AME 0.10, AME–ALE 0.11, PME–PME 0.13, PME–PLE 0.21, ALE–PLE 0.03. MOA 0.57 long, front width 0.49, back width 0.51. Clypeus height 0.32. Chelicerae brown, with three promarginal and three retromarginal teeth. Leg measurements: I 18.62 (4.84, 6.42, 5.21, 2.15); II 16.57 (4.46, 5.55, 4.62, 1.94); III 10.97 (3.35, 3.50, 2.86, 1.56); IV 13.24 (3.94, 3.99, 3.67, 1.64). Leg formula: 1243.

***Palp*** (Figs 4A–D). Patella as long as tibia, ventrally grooved, dorsally with long thick spine. Tibia cone-shaped, with two retrolateral and one dorsal trichobothria, retrolateral margin with nine thick spines. Cymbium with an ectal process wider than long, half the length of tibia, with bifurcated tip, both rami are almost equal in size and shape with blunt end; retrolateral margin of cymbium with thumb-shaped projection extending ventrally with blunt tip. Paracymbium bowl-shaped, apically hook-shaped, with median margin edge curved inward. Tegulum large, apically tapering. Conductor membranous, almost half the length of patella, present on apical end of tegulum. Embolus circular with fine tip, embolic flap horn-shaped, curved with pointed tip, embolic process sclerotized, apically with pointed tip, extending towards ventral side of tegulum.

**Female** (paratype, Fig. 6E) total length 9.64. Prosoma 3.66 long, 2.94 wide; opisthosoma 6.09 long, 4.88 wide. Eye sizes and interdistances: AME 0.23, ALE 0.25, PME 0.23, PLE 0.23; AME–AME 0.08, AME–ALE 0.16, PME–PME 0.16, PME–PLE 0.20. ALE–PLE 0.02. MOA 0.65 long, front width 0.50, back width 0.60. Clypeus height 0.32. Leg measurements: I 15.87 (442, 5.34, 4.04, 2.07); II 14.46 (4.09, 4.76, 3.70, 1.91); III 10.96 (325, 3.53, 2.77, 1.41); IV 13.62 (3.92, 4.37, 3.42, 1.91). Leg formula: 1243.

***Epigyne*** (Fig. 5A–D). Epigynal plate wider than long. Most of the atrium divided by septum. Ventral plate oval, anteriorly grooved, posterior margin wavy. Copulatory openings present within atrium. Dorsal plate somewhat triangular, with broad tip posteriorly. Copulatory ducts sinuous, forming three curved loops before entering spermathecae. Spermathecae round, separated by distance equal to three times their diameter. Fertilization ducts present mesally.

##### Variation.

Males (*N* = 23) total length 6.26–7.87; females (*N* = 15) total length 7.37–9.64.

##### Distribution.

Known only from the type locality, Sichuan, China (Fig. 7).

## Supplementary Material

XML Treatment for Weintrauboa

XML Treatment for Weintrauboa denticulata

XML Treatment for Weintrauboa shenwu

XML Treatment for Weintrauboa wanglangensis

## ﻿Additional information

### Author ORCIDs

Zhizhong Gao https://orcid.org/0000-0002-6666-8746

Muhammad Irfan https://orcid.org/0000-0003-0445-9612

Lu-Yu Wang https://orcid.org/0000-0002-5250-3473

### Data availability

All of the data that support the findings of this study are available in the main text.

## References

[B1] Hormiga G (1994) Cladistics and the comparative morphology of linyphiid spiders and their relatives (Araneae, Araneoidea, Linyphiidae). Zoological Journal of the Linnean Society 111: 1–71. 10.1111/j.1096-3642.1994.tb01491.x

[B2] Hormiga G (2008) On the spider genus *Weintrauboa* (Araneae, Pimoidae), with a description of a new species from China and comments on its phylogenetic relationships. Zootaxa 1814: 1–20. 10.11646/zootaxa.1814.1.1

[B3] Hormiga G Kulkarni S da Silva Moreira T Dimitrov D (2021) Molecular phylogeny of pimoid spiders and the limits of Linyphiidae, with a reassessment of male palpal homologies (Araneae, Pimoidae). Zootaxa 5026(1): 71–101. 10.11646/zootaxa.5026.1.3 34810940

[B4] Irfan M Peng XJ (2018) Three new species of Linyphiidae (Arachnida: Araneae) from Yunnan, China. Oriental Insects 52(3): 229–244. 10.1080/00305316.2017.1398115

[B5] Irfan M Peng XJ (2019a) *Herbiphantes* Tanasevitch, 1992 and *Labullinyphia* van Helsdingen, 1985 (Araneae, Linyphiidae), two newly recorded spider genera from the Gaoligong Mountains in China with the description of two new species. Zootaxa 4638(4): 547–561. 10.11646/zootaxa.4638.4.5 31712460

[B6] Irfan M Peng XJ (2019b) The genus *Parbatthorax* Tanasevitch, 2019 (Araneae, Linyphiidae) new to China, with a new species from the Gaoligong Mountains. European Journal of Taxonomy 555: 1–19. 10.5852/ejt.2019.555

[B7] Irfan M Zhou GC Peng XJ (2019) *Zhezhoulinyphia* gen. nov. (Araneae, Linyphiidae) from Yunnan, China. ZooKeys 862: 43–60. 10.3897/zookeys.862.31406 31341385 PMC6635383

[B8] Irfan M Zhou GC Bashir S Mukhtar MK Peng XJ (2020) *Yuelushannus* gen. nov. (Araneae, Linyphiidae) from China. European Journal of Taxonomy 642: 1–17. 10.5852/ejt.2020.642

[B9] Irfan M Bashir S Peng XJ (2021) *Acroterius* gen. nov. (Araneae: Linyphiidae: Linyphiinae) with twelve new species from Yunnan, China. European Journal of Taxonomy 743: 1–53. 10.5852/ejt.2021.743.1293

[B10] Irfan M Wang LY Zhang ZS (2022a) Two new species of Micronetinae Hull, 1920 spiders (Araneae: Linyphiidae) from Yintiaoling Nature Reserve, Chongqing, China. Acta Arachnologica Sinica 31(1): 17–26. 10.3969/j.issn.1005-9628.2022.01.003

[B11] Irfan M Zhang ZS Peng XJ (2022b) Survey of Linyphiidae (Arachnida: Araneae) spiders from Yunnan, China. Megataxa 8(1): 1–292. 10.11646/megataxa.8.1.1

[B12] Irfan M Wang LY Zhang ZS (2023a) One new genus and nine new species of Linyphiidae spiders from Yintiaoling Nature Reserve, Chongqing of China. Zootaxa 5257(1): 82–114. [incl. Erratum: Zootaxa 5263(4): 575–600.] 10.11646/zootaxa.5257.1.7 37044967

[B13] Irfan M Wang LY Zhang ZS (2023b) Survey of Linyphiidae spiders (Arachnida: Araneae) from Wulipo National Nature Reserve, Chongqing, China. European Journal of Taxonomy 871: 1–85. 10.5852/ejt.2023.871.2129

[B14] Irfan M Dai Y Wang LY Zhang ZS (2024) Four new species of *Tapinocyba* Simon, 1884 (Araneae, Linyphiidae) from Jiangjin District of Chongqing, China. ZooKeys 1219: 195–214. 10.3897/zookeys.1219.133899 39822852 PMC11736462

[B15] Irfan M Zhou GC Peng XJ Zhang ZS (2025) Survey of Linyphiidae spiders (Arachnida: Araneae) from some oriental regions of China. Megataxa 15(1): 1–248. 10.11646/megataxa.15.1.1

[B16] Shorthouse DP (2010) SimpleMappr, an online tool to produce publication-quality point maps. https://www.simplemappr.net [Accessed on 07 January 2025]

[B17] Tanasevitch AV (2025) Linyphiidae Spiders of the World. http://old.cepl.rssi.ru/bio/tan/linyphiidae/ [Accessed on, 05 January 2025]

[B18] WSC (2025) World Spider Catalog. Version 25.5. Natural History Museum Bern, online at http://wsc.nmbe.ch accessed on 07 January 2025. 10.24436/2

[B19] Yang L Yao ZY Irfan M He QQ (2023) A newly recorded genus with description of a new cave-dwelling species of *Flagelliphantes* (Araneae, Linyphiidae) from northeastern China. Biodiversity Data Journal 11: e105488. [1–7] 10.3897/BDJ.11.e105488 PMC10242402 37288000

[B20] Zhang MT Liu P Irfan M Peng XJ (2022) A survey of the genus *Himalaphantes* Tanasevitch, 1992 (Araneae, Linyphiidae) with description of three new species from Yunnan, China. ZooKeys 1123: 47–62. 10.3897/zookeys.1123.86261 36762043 PMC9836660

[B21] Zhao QY Li SQ (2014) A survey of linyphiid spiders from Xishuangbanna, Yunnan Province, China (Araneae, Linyphiidae). ZooKeys 460: 1–181. 10.3897/zookeys.460.7799 PMC4283641 25561858

[B22] Zhou GC Irfan M Peng XJ (2018) Redescription of *Ketambeanigripectoris* (Oi, 1960) comb. nov. (Araneae: Linyphiidae). Turkish Journal of Zoology 42(4): 488–494. 10.3906/zoo-1803-29

[B23] Zhou GC Irfan M Peng XJ (2021) A new species of *Denisiphantes* Tu, Li & Rollard, 2005 (Araneae, Linyphiidae) from Yunnan, China. ZooKeys 1023: 1–12. 33776511 10.3897/zookeys.1023.62025 PMC7969583

[B24] Zhou GC Du WF Xu CX Irfan M (2023) A new species of *Floronia* Simon, 1887 from Baiyan Cave in Guizhou Province, China (Araneae, Linyphiidae). ZooKeys 1185: 309–319. 10.3897/zookeys.1185.109285 38074907 PMC10709813

